# 1545. Evaluation of Pre-exposure Prophylaxis Re-engagement Outreach Initiative for the Prevention of Human Immunodeficiency Virus Transmission

**DOI:** 10.1093/ofid/ofad500.1380

**Published:** 2023-11-27

**Authors:** Suzanne Molino, Justin Ramnarain

**Affiliations:** Gilead Sciences, Brooklyn, New York; Long Island University, Brooklyn, New York

## Abstract

**Background:**

Pre-exposure prophylaxis (PrEP) is an effective method for preventing Human Immunodeficiency Virus (HIV) transmission through sex and injection drug use. However, studies have shown that HIV prevention services are limited in underserved populations. Nationally in non-research settings, data estimates 15-62% PrEP retention in care. At our clinic, approximately 15% of patients were retained in PrEP care between 2018 and 2022, with limited data on reasons why. The objective of this study is to provide data on PrEP patients lost to follow-up.

**Methods:**

This was a single-center, retrospective, descriptive study of PrEP patients seen at the clinic who received at least one PrEP prescription and were lost to follow-up between September 2018 and June 2022. Between July and December 2022, patients lost to follow-up were contacted via telephone to re-engage in care and administered a patient questionnaire. Patients were included if they were 18 years or older, at high risk for HIV acquisition per the Centers for Disease Control, and were lost to follow up for PrEP services, defined as at least three months' time from last clinic visit. Patients were excluded if they had a previous HIV diagnosis or a clinic encounter for HIV post-exposure or HIV/sexually transmitted infection screening only. Endpoints included baseline demographics for all patients and questionnaire results from those who consented to participate. Data collection via EPIC™ chart review and statistical analysis via Microsoft Excel. Descriptive statistics were used to present results.

**Results:**

Sixty-one patients were included. Patients had an average age of 37 years, 70% were male, and 77% were Black/African American. Most patients engaged in high-risk heterosexual behavior with multiple sexual partners of unknown HIV status and inconsistent condom use. Thirty-five patients participated in the questionnaire. Twenty-four patients endorsed self-discontinuing PrEP therapy due to: medication cost/access (6/24); difficulty adhering to clinic visits (8/24); changes to perceived risk of HIV transmission (9/24).

**Figure:**
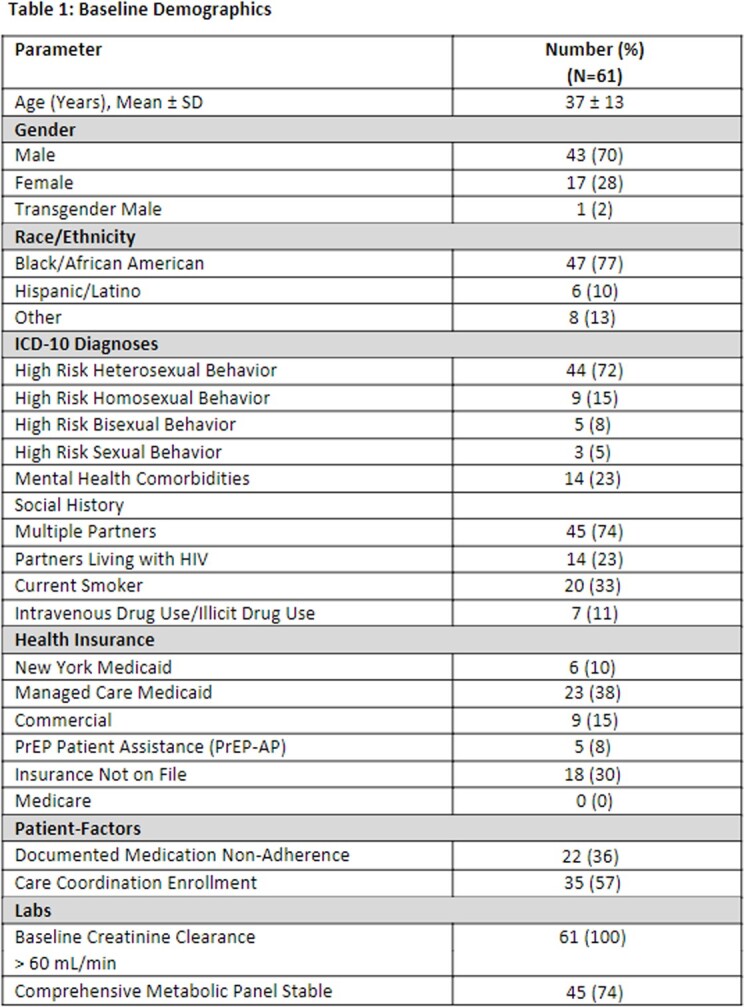

**Figure:**
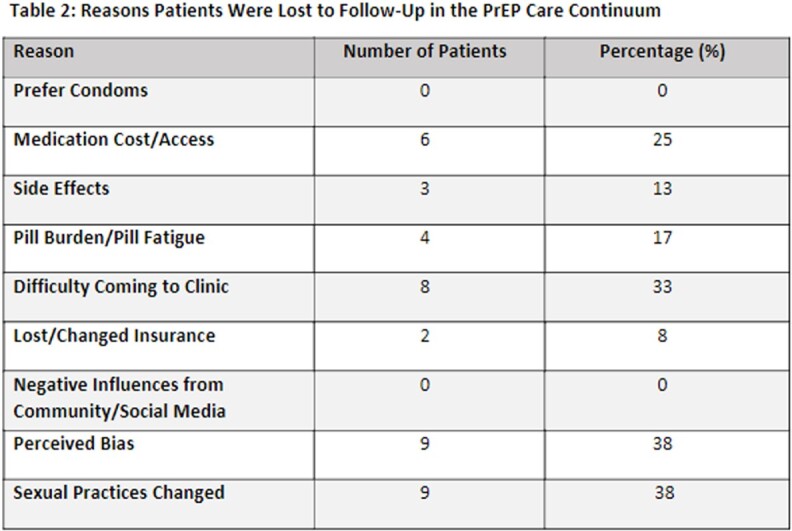

**Conclusion:**

Medication cost/access, adherence to clinic visits, and perceived risk were the top three reasons for PrEP discontinuation. Further research is needed to address barriers to retaining PrEP patients in care.

**Disclosures:**

**All Authors**: No reported disclosures

